# Real-world efficacy of oral upadacitinib for vitiligo and factors associated with treatment response: A retrospective study

**DOI:** 10.1016/j.jdin.2026.04.016

**Published:** 2026-05-04

**Authors:** Yuexi Liu, Xiaoyi Shi, Yukun Yuan, Rongsi Sun, Juan Du, Fang Wang, Xiaolan Ding

**Affiliations:** Department of Dermatology, Peking University People’s Hospital, No. 11 Xizhimen South Street, Xicheng District, Beijing, China

**Keywords:** effectiveness, Janus kinase inhibitor, safety, upadacitinib, vitiligo

*To the Editor:* Vitiligo is a chronic, autoimmune depigmented disorder, and the management of vitiligo remains challenging.^1^ Upadacitinib, an oral selective Janus kinase (JAK) 1 inhibitor, demonstrated favorable efficacy in patients with nonsegmental vitiligo (NSV) in a phase II trial.^2^ However, evidence regarding the performance of upadacitinib in complex, real-world settings remains scarce.

This retrospective study included 53 patients with vitiligo treated with upadacitinib for at least 3 months in our hospital from December 2023 to November 2025. Disease severity was measured using the Vitiligo Area Scoring Index (VASI). The primary outcome of the study was the percentage change in total-VASI (T-VASI) and facial-VASI (F-VASI) between the onset of upadacitinib treatment and the end point for our data collection.

Characteristics of all patients are summarized in Table I. The mean (SD) age of participants was 32.96 ± 15.27 years; 29 (54.7%) were female. Most patients (81.1%) were in the progressive stage with a vitiligo disease activity (VIDA) score ≥3. The patients received upadacitinib 15 mg per day with a median duration of 4 months (range 3 to 16 months).

**Table I:** Demographic and clinical characteristics of all patients with vitiligo

Variables	Results (*n* = 53)
Age, y	
Mean (SD)	32.96 (15.27)
Adults (≥18 y), *n* (%)	42 (79.3)
Sex, *n* (%)	
Female	29 (54.7)
Male	24 (45.3)
BMI, kg/m^2^, median (IQR)	22.86 (20.75-24.86)
Total duration of disease, y	
Median (IQR)	4.00 (1.50-10.00)
<10 y, *n* (%)	33 (62.3)
≥10 y, *n* (%)	20 (37.7)
VIDA score, median (IQR)	4.00 (3.00-4.00)
Vitiligo subtype, *n* (%)	
NSV	52 (98.1)
SV	1 (1.9)
Severity of disease (BSA grading), *n* (%)	
Mild to moderate	40 (75.5)
Severe	13 (24.5)
Lesion involvement, *n* (%)	
Face	27 (50.9)
Trunk	36 (67.9)
Extremities	35 (66.0)
Hand and/or foot	37 (69.8)
Vitiligo-associated leukotrichia, *n* (%)	9 (17.0)
Comorbidities, *n* (%)	
Atopic dermatitis	7 (13.2)
Autoimmune thyroid diseases	3 (5.7)
Other diseases∗	3 (5.7)
Family history of vitiligo, *n* (%)	8 (15.1)
History of prior treatment, *n* (%)	
Systemic glucocorticoids	39 (73.6)
Traditional Chinese medicine†	35 (66.0)
Topical treatment	44 (83.0)
Phototherapy	34 (64.2)
Oral JAK inhibitors‡	11 (20.8)
Surgical modalities	4 (7.6)
Baseline BSA, %, median (IQR)	2.08 (0.70-8.59)
Baseline T-VASI score, median (IQR)	2.03 (0.60-8.59)
Baseline F-VASI score (*n* = 27), median (IQR)	0.1 (0.03-0.30)
Duration of treatment with upadacitinib, m	
Median (IQR)	4.00 (3.00-6.00)
3 m ≤ T < 6 m, *n* (%)	35 (66.0)
T ≥ 6 m, *n* (%)	18 (34.0)
Combination treatment, *n* (%)	
None	6 (11.3)
Topical treatment	38 (71.7)
Phototherapy	32 (60.4)

*BMI*, Body mass index; *BSA*, body surface area; *F-VASI*, Facial Vitiligo Area Scoring Index; *JAK*, Janus kinase; *NSV*, nonsegmental vitiligo; *SV*, segmental vitiligo; *T-VASI*, Total Vitiligo Area Scoring Index; *VIDA*, vitiligo disease activity.

∗ Other diseases include eczema, chronic urticaria, and endometriosis.

† Indicates Chinese herbs or traditional Chinese patent medicine, including compound glycyrrhizin, Bailing tablet, and Baidianfeng capsule.

‡ Oral JAK inhibitors include baricitinib and tofacitinib.

After treatment with oral upadacitinib, the median (IQR) T-VASI and F-VASI score dropped from 2.03 (0.60-8.59) and 0.1 (0.03-0.30) at baseline to 1.11 (0.39-3.70) and 0.03 (0.00-0.10), respectively, both indicating a significant reduction (*P* < .001) with a median improvement of 22.3% (range 2.1% to 94.6%) and 66.7% (range 10% to 100%). Overall, 26 (49.1%) patients achieved T-VASI25 and 11 (20.8%) achieved T-VASI50. Among the 27 patients with facial vitiligo, 19 (70.4%) achieved F-VASI50 and 9 (33.3%) achieved F-VASI90. Among the 35 patients with distal extremity involvement, response rates varied from 0% to 95%. Treatment outcomes are shown in Table II.

**Table II:** Outcomes of efficacy after upadacitinib treatment

Variables	Results
Outcomes of efficacy indicators	
T-VASI	
Improvement, median (IQR), %	22.3 (10.1-42.4)
T-VASI25, *n* (%)	26 (49.1)
T-VASI50, *n* (%)	11 (20.8)
F-VASI	
Improvement, median (IQR), %	66.7 (33.3-100.0)
F-VASI50, *n* (%)	19 (70.4)
F-VASI90, *n* (%)	9 (33.3)
Distal extremities (hand/foot) improvement, median (IQR), %	10.0 (0.0-20.0)
Leukotrichia improvement, *n*, %	2 (22.2)
BSA improvement, median (IQR), %	18.2 (9.5-29.2)
Repigmentation type, *n* (%)	
Perifollicular repigmentation	39 (73.6)
Marginal repigmentation	15 (28.3)
Uniform repigmentation	2 (3.8)
Onset of repigmentation during treatment, *n* (%)∗	
Within mo 1	44 (83.0)
Within mo 2	6 (11.3)
Within mo 3	3 (5.7)

*F-VASI*, Facial Vitiligo Area Scoring Index; *T-VASI*, Total Vitiligo Area Scoring Index; *T-VASI25, T-VASI50, F-VASI50, F-VASI90*, the proportions of patients achieving at least 25%, 50% improvement from baseline T-VASI and 50%, 90% improvement from baseline F-VASI.

∗ This is the time recording when repigmentation was first recognized during the treatment of upadacitinib.

Multivariate analysis showed that combination treatment with topical drugs (β = 14.66, 95% CI = 0.84-28.28, *P* = .038) was statistically associated with improved effectiveness (Supplementary Table I, available via Mendeley at https://data.mendeley.com/datasets/2j8ybp4w5c/1). Additionally, subgroup analysis revealed higher T-VASI response rates in the mild-moderate group vs the severe group (28.7% vs 10.5%, *P* = .032), and in the JAK inhibitor-naïve group versus the JAK inhibitor-experienced group (28.7% vs 10.0%, *P* = .032).

Mild to moderate adverse reactions were reported in 17 (32.1%) patients, with acne (18.9%) and upper respiratory tract infection (11.3%) being the most frequent (Supplementary Table II, available via Mendeley at https://data.mendeley.com/datasets/2j8ybp4w5c/1). No serious adverse events occurred throughout the study period.

This real-world study demonstrated the efficacy and safety of oral upadacitinib in a diverse cohort of vitiligo patients. Consistent with previous findings,^3^ facial lesions showed pronounced repigmentation while distal extremities exhibited minimal enhancement. A recent study reported notable repigmentation in progressive NSV patients treated with upadacitinib plus 1.5% ruxolitinib cream.^4^ Our findings indicated concomitant use of topical tacrolimus or corticosteroids as a significant factor of superior efficacy, suggesting that oral upadacitinib combined with topical therapy is likely to be a promising strategy for vitiligo. Nevertheless, diminished efficacy was noted in patients with severe disease or prior JAK inhibitor exposure. The study has some limitations, including a limited sample size, variability in treatment duration, and relatively short follow-up time. Further large-scale, prospective studies are needed to validate our findings and evaluate the long-term efficacy of upadacitinib in vitiligo.

## Conflicts of interest

None disclosed.
